# Impact of soybean meal enzymatic treatment on chicken caecal microbiota and intestinal epithelial cells

**DOI:** 10.1007/s00253-026-13925-8

**Published:** 2026-06-23

**Authors:** Lauriane Plouhinec, Marc Maresca, Julie Saget, Virginie Neugnot, Estelle Devillard, Jean-Guy Berrin, Mickael Lafond

**Affiliations:** 1 Aix-Marseille Université, INRAE, BBF, Biodiversité et Biotechnologie Fongiques, Marseille, 13009 France; 2 Aix-Marseille Université, CNRS, Centrale Marseille, iSM2, Marseille, 13013 France; 3 Adisseo France S.A.S., European R&I Centre ELISE, Saint-Fons, 69190 France; 4 Adisseo France S.A.S, CINAbio, INSA Toulouse, Toulouse, 31400 France

**Keywords:** Chicken intestinal health, Fungal secretomes, Enzymes, CAZymes, Pectin, Soybean meal, Short-chain fatty acids

## Abstract

**Abstract:**

As demand for sustainable and welfare-conscious food production increases, reducing the environmental and health impacts of animal feed has become a key challenge. Soybean meal (SBM) is widely used in poultry diets for its high protein content, but its richness in non-starch polysaccharides (NSPs) can hamper its digestibility. To address this issue, a common strategy involves the utilization of enzymatic cocktails rich in carbohydrate-active enzymes (CAZymes). While these feed additives are specifically designed to degrade NSPs and enhance SBM protein digestibility, only scarce information is available on their impact on intestinal health. In this study, using intestinal epithelial cells lines, we show that pre-treatment of SBM by the enzymatic cocktail Rovabio™ Advance, supplemented or not with pectin-active fungal secretomes, did not alter epithelial integrity nor induce inflammatory responses. Additionally, in vitro chicken caecal fermentations revealed that SBM was associated with a higher relative abundance of *Enterobacteriaceae*, while wheat fermentation was associated with *Lactobacillaceae* and *Enterococcaceae* enrichment. Enzymatic supplementation with Rovabio™ Advance further enhanced wheat fermentation and bacterial community shifts, while its effect on SBM fermentation remained limited. In contrast, SBM pre-treatment with pectin-active fungal secretomes led to significantly increased short-chain fatty acid (SCFA) production and enriched SCFA-associated genera. These findings indicate that while industrial enzyme cocktails are well suited to cereal-based diets, targeted pectin-degrading enzymes could be seen as a promising strategy to enhance the prebiotic potential of SBM. This study highlights the importance of tailoring enzymatic solutions to feed carbohydrate composition, to improve gut microbiota function and promote intestinal health in poultry.

**Key points:**

• *SBM does not induce intestinal cell inflammation or epithelium disruption in vitro.*

• *Both SBM and wheat increase SCFA production by the chicken caecal microbiota.*

• *Pre-treatment with fungal pectin-active secretomes enhance SBM’s prebiotic potential.*

**Supplementary Information:**

The online version contains supplementary material available at https://doi.org/10.1007/s00253-026-13925-8.

## Introduction

As the global human population continues to increase, food demand is projected to rise faster than changes in dietary habits and consumption patterns. While interest in sustainable and animal-welfare-conscious food production is growing in Western countries (Ammann et al. 2024), demand for meat and animal products in lower middle-income countries is expected to increase steadily over the next decade. To meet this demand, global agricultural production would need to rise by about 14%, with direct greenhouse gas emissions from agriculture projected to increase by 6% by 2034 (OECD/FAO 2025). Within this context, environmental and economic considerations position poultry as the primary global meat source, with poultry meat predicted to provide nearly half of all protein derived from meat within the next ten years (OECD/FAO 2025). The chicken gut microbiota plays an important role in feed utilization, animal health and productivity, making it a key target for nutritional strategies in modern poultry production (Jha et al. 2019). Current research on chicken microbiota shows that the caecum hosts the most complex and diverse microbial community within the gut (Feng et al. 2023). One of the main roles of the chicken caecal microbiota is to ferment undigested compounds such as dietary non-starch polysaccharides (NSPs), leading to the production of short-chain fatty acids (SCFAs) that are metabolised by the host and contribute to physiological functions such as strengthening gut barrier function and reducing intestinal inflammation (Liu et al. 2021). The chicken microbiota is influenced by several factors, particularly feed composition and antibiotic use. Additionally, breeding conditions (e.g., intensive vs. semi-wild) have been shown to affect bacterial populations (Mancabelli et al. 2016; Cheng et al. 2025). To study these changes, commonly used techniques include metabarcoding by 16S DNA or RNA sequencing and metabolomics through the quantification of SCFA. The chicken microbiota is predominantly composed of bacteria from the *Firmicutes* phylum, with the families *Ruminococcaceae*, *Lactobacillaceae* and *Lachnospiraceae* being the most abundant (Burrows et al. 2025). It is also known that the majority of carbohydrates-active enzymes (CAZymes) found in the chicken gut are glycoside hydrolases targeting starch and cellulose (Segura-Wang et al. 2021), which align with the diet of broiler chicken that mainly consists of cereals (Adebowale et al. 2019). Nevertheless, this enzymatic diversity remains limited, and key hemicellulases and pectinases needed to fully degrade these complex carbohydrate-rich meals are still largely underrepresented (Feng et al. 2021; Segura-Wang et al. 2021; Plouhinec et al. 2023; Shen et al. 2024).

Over the past 50 years, bacterial resistance to antibiotics has become a major public health concern for both humans and animals (Verraes et al. 2013; Shen et al. 2018; Iwu et al. 2020; Nadgir and Biswas 2023). One strategy to prevent antibiotics resistance is to reduce their environmental release, which has led the European Union and some other countries to ban their use for preventive and growth-promoting purposes in animal breeding (European Commission 2005; Agyare et al. 2018). Since then, the concept of “health-by-nutrition” has risen in the animal feed industry, making intestinal health and microbiota balance key economic focuses for poultry producers (Choct 2009). Today, several alternatives have been reported in literature in order to maintain animal performances, one of them being the use of NSP-degrading enzymes (Abd El-Hack et al. 2022; Plouhinec et al. 2023). These enzyme-based solutions improve diet digestibility, reducing bolus viscosity while potentially releasing oligosaccharides with prebiotic effect from agricultural products and co-products used in animal feed (Reis et al. 2014; Chimtong et al. 2016; Prandi et al. 2018; Valladares-Diestra et al. 2023). One of the most widespread agricultural co-products in animal feed is soybean meal (SBM), used as a main protein source for broiler chickens.

In addition to its high content in proteins, SBM also contains compounds known as antinutritional factors (ANFs). While some of them are inactivated by toasting during processing, some heat-stable ANFs remain, such as certain polysaccharides (Lambo et al. 2023). Incomplete degradation of these compounds in the upper digestive tract can lead to reduced nutrient absorption and increased fibre fermentation by the gut microbiota (Singh and Kim 2021). Given the complexity of SBM carbohydrates and the diversity of the chicken gut microbiota, predicting the impact of SBM on intestinal health can be challenging. Indeed, SBM contains substantial amounts of NSPs, mainly hemicelluloses and pectin (Knudsen 2014). Studies about the impact of pectin on intestinal health alternatively describe it as both a friend and a foe. While the solubility of pectin tends to form gels, increasing feed viscosity and slowing down nutrient assimilation (Musigwa et al. 2021), several studies described the prebiotic activity of pectin oligosaccharides in human and animal gut (Babbar et al. 2016; Chung et al. 2017; Míguez et al. 2020; Wang et al. 2022). Therefore, the enzymatic degradation of SBM could both reduce its ANFs content and reveal its beneficial effects on intestinal health, through the release of prebiotic-like compounds. In that context, enzymatic cocktails containing CAZymes have been used for many years in animal feed (Plouhinec et al. 2023), one of them being the Rovabio™ Advance, produced by the fermentation of *Talaromyces versatilis*. While its efficacy in cereal-based diets has been extensively demonstrated and optimized (Maisonnier-Grenier et al. 2006; Lafond et al. 2011, 2015; Cozannet et al. 2017, 2019; Guais et al. 2017), previous studies have shown that SBM pectin hydrolysis by Rovabio™ Advance can be further improved, particularly through supplementation with *Aspergillus terreus* secretomes (Grandmontagne et al. 2021; Plouhinec et al. 2024).

In the present study, we investigated the impact of enzymatically treated SBM on intestinal health in vitro. Using different enzymatic cocktails, including Rovabio™ Advance and pectin-active fungal secretomes, we aimed to evaluate how SBM degradation products influence intestinal inflammation and microbial fermentation in chickens. Experiments on enterocyte cell lines were conducted to assess inflammatory responses to SBM with and without enzymatic pre-treatment. This approach was complemented by in vitro fermentations using chicken caecal contents to characterise microbial activity through SCFA quantification and analysis of bacterial diversity by 16S DNA metabarcoding. Microbial fermentations of SBM and wheat were compared to assess the impact of these two major components of poultry diets on chicken gut microbiota, with and without enzymatic treatment with Rovabio™ Advance. Finally, the impact of pectin-active fungal secretomes was evaluated to better understand how enzymatic processing of feed ingredients contributes to gut health through microbiota modulation.

## Methods

### Materials

Unless stated otherwise, materials were purchased from Thermo Fisher Scientific® (Waltham, MA, USA). Human IL-1β was purchased from PeproTech® (ref 200-01B-10UG, Cranbury, NJ, USA). Porcine IL-1β was purchased from R&D systems® (ref 681-PI, Minneapolis, MN, USA). Enzyme-linked immunosorbent assay (ELISA) kit for human IL-8 quantification was purchased from BD Biosciences® (ref 555,244; Franklin Lakes, NJ, USA). ELISA kit for porcine IL-8 quantification was purchased from Invitrogen® (ref KSC0081; Waltham, MA, USA). Soybean meal (SBM) and wheat were provided by the Centre of Expertise and Research in Nutrition (CERN, Commentry, France) of Adisseo®. SBM is composed of an equal dry weight ratio of several SBM batches from SojaProtein (SOPRO UTG and GRIT48, Serbia), from Terrena (France), and from BRF-NovaMutum (Brazil—Midwest), all ground to a thickness of 3 mm. Wheat originates from Société Michel (France, Teexma: 23.M.000338) with a thickness of 1 mm. Rovabio™ Advance was also provided by the CERN. The same batch of the liquid concentrated form, stabilized with 0.35% (w/w) of sodium benzoate, was used for all hydrolysate preparations. Fungal secretomes were obtained by cultivating *A. terreus* CIRM-BRFM 111 or *T. versatilis* IMI 378536 on sugar beet pulp and harvesting culture supernatants at day 3, as previously described (Plouhinec et al. 2024).

### Experimental design

To evaluate the effects of SBM on chicken intestinal health, two in vitro models were used: intestinal epithelial cell lines and chicken caecal microbiota (Supplementary Fig. S1). The first model provides insights into the toxic and inflammatory effects of SBM, while the second assesses its impact on gut microbial communities. Several enzymatic treatments were applied to SBM, including (i) Rovabio™ Advance alone, (ii) fungal secretomes alone and (iii) Rovabio™ Advance + fungal secretomes. For the intestinal cell lines assays, two cell models were selected: Caco-2 and IPEC-J2, for their ability to form polarised monolayers with tight junctions and to secrete cytokines (Vergauwen 2015; Ponce de León-Rodríguez et al. 2019; Maresca et al. 2021). Although Caco-2 is a human intestinal model derived from cancerous colorectal carcinoma, it is widely used as a reference for absorption and permeability assays, particularly for drug and chemical assimilation studies (Rao and Sankar 2009; Angelis and Turco 2011). On the other hand, IPEC-J2 is a porcine intestinal model isolated from the jejunum of healthy piglets (Brosnahan and Brown 2012), which is even more relevant for evaluating the effects of SBM on intestinal health in monogastric animals. Both cell models were exposed to increasing doses of SBM, either untreated or treated with fungal secretomes and Rovabio™ Advance, under the conditions listed in Supplementary Table S1. To evaluate the effect of feed component on the chicken caecal microbiota, fermentations were performed with wheat as positive control, as previously described (Yacoubi et al. 2016), for comparison with SBM. To evaluate the effect of enzymatic treatment of SBM, five different treatments were applied to SBM, as listed in Supplementary Table S2.

### Preparation of SBM hydrolysates

SBM and wheat were autoclaved at 110 °C for 30 min prior to enzymatic treatment to avoid any bacterial or fungal contamination. For intestinal cells lines assays, 75 mg of autoclaved SBM was treated with or without enzymatic cocktails for 48 h at 37 °C in 1 mL sodium acetate buffer (50 mM, pH 5.2). Reactions were performed in biological replicates (*n* = 3) in 2-mL Eppendorf tubes. After hydrolysis, reactions were stored at + 4 °C before application on the cells. The entire reaction mixture (soluble and insoluble fractions) was used for the assays. The list of experimental conditions tested is described in Supplementary Table S1. For microbial fermentations, 75 mg of autoclaved SBM or wheat were treated with or without enzymatic cocktails for 48 h at 37 °C in 1 mL sodium acetate buffer (50 mM, pH 5.2). Reactions were performed in biological replicates (*n* = 4) in 2-mL Eppendorf tubes. After hydrolysis, the entire reaction mixture (soluble and insoluble fractions) was transferred into sterile 12-mL Hungate tubes and freeze-dried at − 60 °C for 48 h, prior to microbial in vitro fermentation. The list of experimental conditions tested is described in Supplementary Table S2.

### Intestinal cells lines culture and maintenance

Intestinal cells were cultivated as previously described (Zhang et al. 2020; Roblin et al. 2021). Caco-2 cells (obtained from ATCC) were maintained in Dulbecco’s Modified Essential Medium (DMEM; Thermo Fisher Scientific®) supplemented with 10% foetal bovine serum and 1% antibiotics (PenStrep, Thermo Fisher Scientific®). IPEC-J2 cells (obtained from DSM) were maintained in DMEM-F12 media supplemented with 10% foetal bovine serum and 1% antibiotics. All cell lines were routinely grown in 75 cm^2^ flasks (Thermo Fisher Scientific®) in a 5% CO_2_ incubator at 37 °C.

### Cytokine quantification and intestinal transepithelial integrity measurements

Cytokine quantification and intestinal transepithelial electric resistance (TEER) measurements were performed as previously described (Rhayat et al. 2019). Cells grown on 75 cm^2^ flasks were detached using trypsin solution, counted using Malassez cell counting, seeded at 100,000 cells/well onto 12-wells inserts (“ThinCert”; 1 cm^2^; pore size 0.4 μm; Greiner Bio-One®, Kremsmünster, Austria) and left to differentiate with media change every 2 days. After a 7-day incubation at 37 °C and 5% CO_2_, inserts and wells were emptied and filled with fresh media in the apical and basolateral compartments before treatment with the samples or controls. To validate the experiments, reference, negative and positive controls were added. Reference control corresponds to cells in culture media (labelled “Untreated” in the “Results” section). Negative controls correspond to enzymatic cocktails and fungal secretomes without SBM. Positive controls correspond to cultured cells of *Salmonella enterica* (CIP 80.39), deoxynivalenol (DON) and recombinant IL-1β (human for Caco-2 and porcine for IPEC-J2), as previously described (Rhayat et al. 2019). Cultured cells of *S. enterica* were added to the apical compartment at a 10^7^ colony forming unit (CFU)/mL final concentration. DON was added to the apical compartment at a 20 µM final concentration. Human or porcine IL-1β were added to the basolateral compartment at 1 µg/mL final concentration. To assess the dose effect, cells were treated with increasing concentrations of SBM hydrolysates, diluted in the appropriate medium. The doses tested ranged from 1:10 to 1:80 dilution in the culture media. After a 12-h exposure to the controls and samples, cell inflammation was measured by quantification of IL-8 secretion in the basolateral media of the inserts using commercial ELISA kits. A 12-h exposure time was selected to reflect poultry digestion time, which averages 4–6 h but may extend up to 12 h. For Caco-2 cells, IL-8 quantification was performed at a 1:5 dilution, while for IPEC-J2 cells, it was done without dilution, to ensure measurements were above the detection threshold and within the standard range. In addition, the impact of the controls and samples on epithelial integrity was measured through determination of the TEER, using Millicell EVOM voltohmmeter (Millipore®, Burlington, MA, USA). These experiments were conducted in biological triplicates.

### In vitro fermentations of chicken caecal contents

Chicken caecal microbial fermentations were performed as previously described (Davies et al. 1995), in the Hungate tubes containing freeze-dried SBM hydrolysates presented earlier. Caecal contents were sampled from Ross 308 male broiler chickens aged 28 days at the CERN and stored at − 80 °C before use. Chickens were fed with a basal diet composed of wheat, corn and SBM without any enzyme supplementation prior to sampling. The fermentation buffer is composed of 5 solutions (A, B, C, D and E) prepared individually: solution A (per litre: 6.2 g KH_2_PO_4_, 3.7 g Na_2_HPO_4_ and 0.6 g MgSO_4_ · 7H_2_O), solution B (per litre: 4 g NH_4_HCO_3_, and 35 g NaHCO_3_), solution C (per litre: 13.2 g CaCl_2_ · 2H_2_O, 10 g MnCl_2_ · 4H_2_O and 8 g FeCl_3_ · 6H_2_O), solution D (0.1% resazurin) and solution E (per 100 mL: 4 mL NaOH 1 M and 655 mg Na_2_S · 9H_2_O, needs to be prepared freshly for each experiment). The fermentation buffer was assembled on the day of the experiment, under anaerobic conditions (using CO_2_ as flushing gas) with 22.2% (v/v) of solution A, 22.2% (v/v) of solution B, 0.01% (v/v) of solution C, 0.1% (v/v) of solution D and 55.5% (v/v) of ultra-pure water (18.2 mΩ). The fermentation buffer was autoclaved for 15 min at 121 °C in the presence of 0.6 g/L of l-cysteine (reducing agent) and was further reduced by addition of 4% (v/v) of solution E on the day of the experiment. The buffer was then incubated in a water bath to let it reach 39 °C before adding the caecal content at 5% (w/v). Once completed, 7.5 mL of the fermentation buffer containing the caecal content was distributed into the Hungate tubes using a peristaltic pump and under anaerobic conditions (using CO_2_ as flushing gas). The tubes were transferred into a water bath set at a temperature of 39 °C and a linear shaking of 150 rpm. After 30 min of incubation, the tubes were degassed, marking the T0 of fermentation. The fermentation was then continued for 24 h with air pressure measurement at 2 h, 4 h, 6 h and 24 h of incubation. After 24 h, the samples were centrifuged at 15,000 g for 15 min to separate the cell pellet from the supernatants. Cells pellets were stored at − 80 °C prior to DNA extraction and 16S amplicon sequencing. The supernatants were aliquoted for pH measurement and SCFA quantification and stored at − 80 °C.

### Quantification of short-chain fatty acids

Acetate, propionate and butyrate were quantified in fermentation supernatants by ionic chromatography using an IonPac AS11 (4 × 250 mm) column (Thermo Fisher Scientific®) with conductimetric detection and external calibration for each SCFA. Samples were prepared by diluting 100 µL of supernatant in 50 mL of ultrapure water prior to injection. Separation was achieved using a KOH gradient at a flow rate of 2 mL/min: 0.20 mmol/L from 0 to 6.5 min, 5.00 mmol/L at 12 min, 38.0 mmol/L at 23 min, and returning to 0.20 mmol/L at 23.5 min until 25 min.

### 16S DNA sequencing and taxonomic classification

Experiments described in this section were performed by BaseClear (Leiden, Netherlands). Genomic DNA was extracted from the cell pellets of caecal fermentation using a commercial extraction kit (KingFisher, Thermo Fisher Scientific®) in presence of DNA/RNA shield. 16S DNA sequencing was carried out by the MiSeq PE300 pair-end sequencing technology (Illumina®, San Diego, CA, USA) for 6 MB/sample. FASTQ read sequence files were generated using bcl-convert version 4.2.4 (Illumina®). Initial quality assessment was based on data passing the Illumina® Chastity filtering. Subsequently, reads containing PhiX control signal were removed using Bowtie2 version 2.4.5 (Langmead and Salzberg 2012). In addition, reads containing (partial) adapters were clipped (up to a minimum read length of 50 bp) using fastp version 0.23.4 (Chen et al. 2018). The second quality assessment was based on the remaining reads using the FASTQC quality control tool version 0.11.9 (Andrews 2010). Illumina® raw sequence data was down sampled using BBmap version 38.90 (Bushnell 2014). Paired-end sequence reads were collapsed into so-called pseudo-reads using sequence overlap with USEARCH version 9.2 (Edgar 2010). Classification of these pseudo-reads was performed based on the results of alignment with SNAP version 1.0.23 (Zaharia et al. 2011) against the RDP database (Cole et al. 2014) version 11.5 for bacterial organisms, or the UNITE ITS gene database (Abarenkov et al. 2010) version 8 for fungal organisms. Illumina sequencing raw data are accessible on the NCBI under the reference PRJNA1465736.

### Bioinformatic and statistical analyses

One-way analysis of variance (ANOVA) followed by Tukey post hoc multiple comparison of means at a 95% family-wise confidence level was applied to the data of SCFA quantification, pressure and pH measurements. Two-way analysis of variance (ANOVA) followed by Tukey post hoc multiple comparison of means at a 95% family-wise confidence level was applied to the data of TEER measurements and of IL-8 quantification, to allow for the analyse of both sample pre-treatment and sample dose. These analyses were done using the R-Studio software for Windows, version 2025.09.2 (Posit Software®, PBC, Boston, MA, USA). Calculations were performed by the R packages stats (version 4.5.2), emmeans (version 2.0.0) and multcompView (version 0.1–10). For the metabarcoding results, statistical analyses were performed using MicrobiomeAnalyst (www.microbiomeanalyst.ca), as previously described (Lu et al. 2023). MicrobiomeAnalyst operates under an R environment to generate plots, diversity indexes and graphs. Specifically, marker data profiling relied on raw Operational Taxonomic Unit (OTU) counts of bacterial families and genera for each sample. For family level analyse, data filtering thresholds included a minimal count of 10 OTUs based on mean abundance value. A low variance filter was applied at 10% based on standard deviation. This data filtering resulted in 17 bacterial families that were considered for further analysis. For genus level analyse, data filtering thresholds included a minimal count of 15 OTUs based on mean abundance value. A low variance filter was applied at 13% based on standard deviation. This data filtering resulted in 50 bacterial genera that were considered for further analysis. Data normalization was achieved through total sum scaling, with no rarefaction applied. Beta-diversity analysis was conducted using Principal Coordinates Analysis (PCoA) based on a Bray–Curtis distance matrix. Pairwise PERMANOVA was employed as the statistical test. For family-level data, linear discriminant analysis (LDA) effect size (LEfSe) comparison of differentially abundant bacterial families was used (Segata et al. 2011). For genus-level data, Multiple Linear Regression with Covariate Adjustment (MaAsLin2) was used, and *p*-values were adjusted to account for multiple testing using the false discovery rate (FDR) method (Mallick et al. 2021).

## Results

### In vitro effect of SBM hydrolysates on transepithelial integrity and cell inflammation

**Fig. 1:**
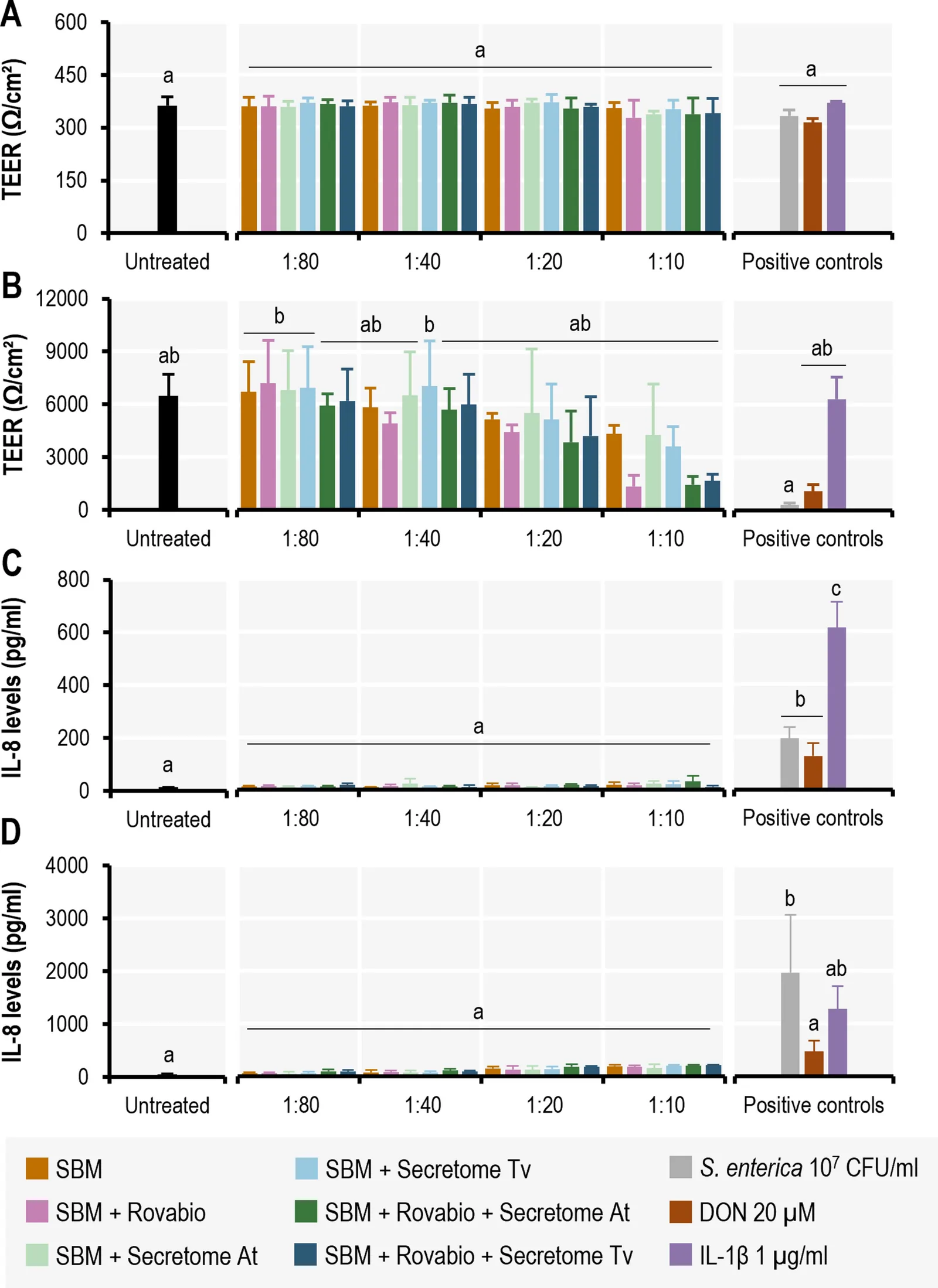
Effect of SBM hydrolysates on in vitro epithelial integrity and cytokine production. (**A**, **B**) Transepithelial electric resistance after 12 h exposure on (**A**) Caco-2 model and (**B**) IPEC-J2 model. (**C**, **D**) IL-8 secretion after 12 h exposure on (**C**) Caco-2 model and (**D**) IPEC-J2 model. Values are presented as means ± SD (biological replicates, *n* = 3). “Untreated” corresponds to the reference control (i.e., cells in culture media). SBM hydrolysates were tested at different dilutions in the culture media (from 1:80 to 1:10) to assess the dose effect. Statistical analyses were performed using two-way ANOVA and Tukey post hoc. Groups indicated by different letters are statistically different (*p* < 0.05). DON: deoxynivalenol; SBM: soybean meal; At: *A. terreus*; Tv: *T. versatilis*

To assess the in vitro effects of enzymatically treated SBM, we first investigated transepithelial integrity and intestinal cell inflammation. Using two cell models (Caco-2 and IPEC-J2), selected for their ability to form polarised monolayers with tight junctions and to secrete cytokines (Vergauwen 2015; Ponce de León-Rodríguez et al. 2019; Maresca et al. 2021), we measured TEER and quantified IL-8 in the basolateral supernatant using ELISA (Fig. 1). Results obtained with negative controls (i.e., enzymatic cocktails and/or reaction buffer alone) are shown in Supplementary Fig. S2. As presented in Fig. 1A and B, untreated IPEC-J2 cells exhibited higher TEER values than untreated Caco-2 cells, consistent with previous studies (Vergauwen 2015). Regardless of enzymatic treatment, SBM did not alter the transepithelial integrity of either Caco-2 or IPEC-J2 cells (Supplementary Table S3), as no significant changes in TEER were observed after 12 h of exposure (Fig. 1A and B). Likewise, exposure to DON, *S. enterica*, or IL-1β at the tested concentrations did not affect TEER. These findings are in line with earlier observations regarding the effect of DON on Caco-2 cells (Maresca et al. 2008), while the absence of an effect in IPEC-J2 cells is most likely related to the shorter exposure time used in this study compared with previous work (Diesing et al. 2012).

In contrast, all positive controls significantly increased IL-8 secretion by Caco-2 cells after 12 h of exposure (Fig. 1C), with up to a 44-fold increase in the presence of human IL-1β compared with untreated cells. In IPEC-J2 cells, 12-h exposure to *S. enterica* similarly induced a significant inflammatory response, as previously reported (Razzuoli et al. 2017). However, as shown in Fig. 1C and D, neither SBM nor enzyme-treated SBM altered IL-8 secretion in Caco-2 or IPEC-J2 cells. Consistent with the absence of TEER changes, these findings indicate that neither untreated nor enzymatically treated SBM induced detectable inflammatory responses in enterocytes in vitro under the tested conditions and across all doses.

### Effect of feed ingredient on chicken caecal microbiota

**Fig. 2:**
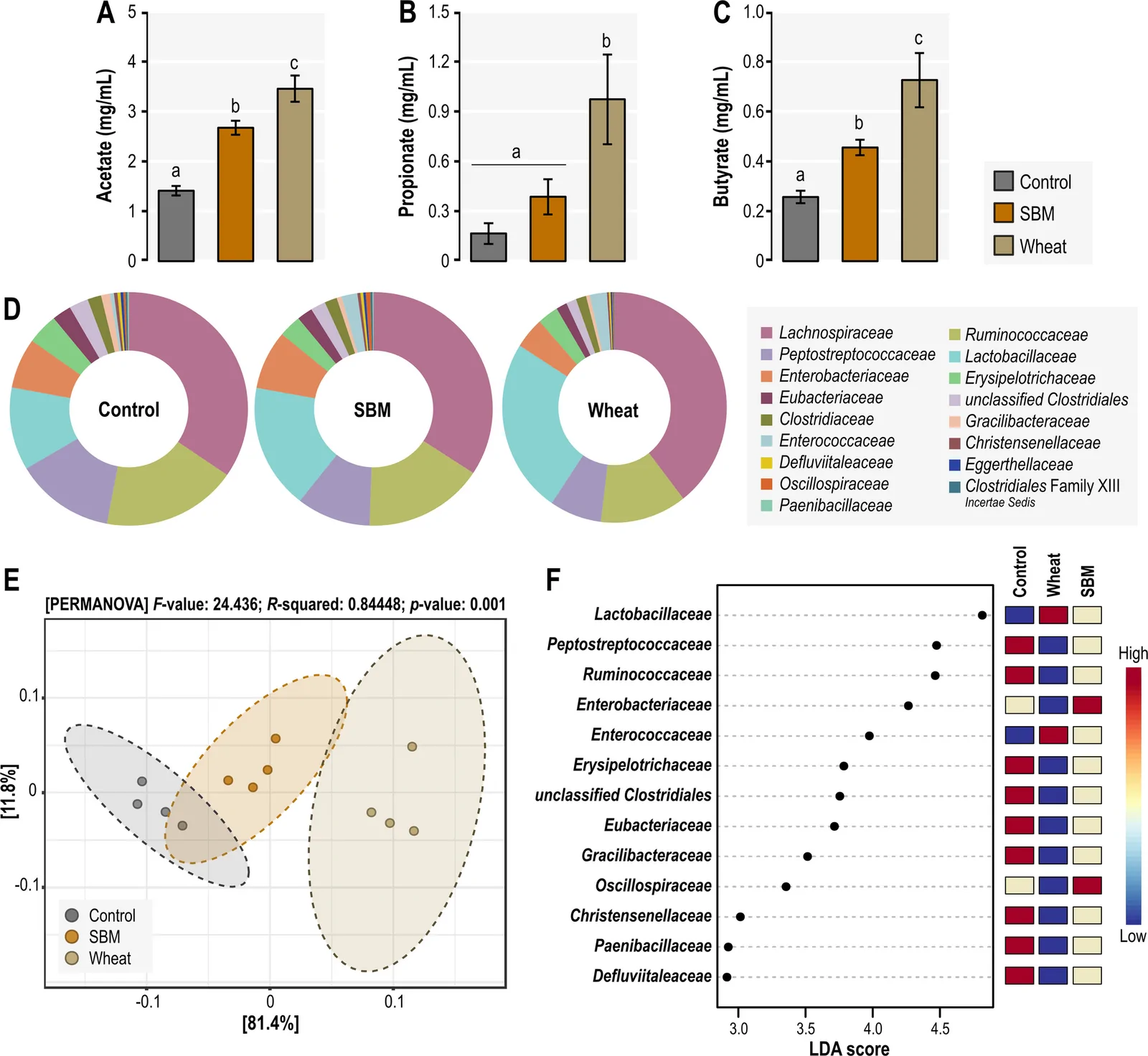
Effect of soybean meal and wheat on chicken caecal microbiota. (**A**–**C**) Concentration of acetate (**A**), propionate (**B**) and butyrate (**C**) in mg/mL in the supernatant after 24-h fermentation. Values are presented as means ± SD (biological replicates, *n* = 4). Statistical analyses were performed using one-way ANOVA and Tukey post hoc. Groups indicated by different letters are statistically different (*p* < 0.01). (**D**) Relative abundance of top 17 bacterial families detected in the samples after data filtering (as described in the “Methods” section). (**E**) Beta-diversity illustrated by Principal coordinate analysis plots using Bray–Curtis dissimilarity. (**F**) Linear discriminant analysis (LDA) effect size (LEfSe) comparison of differentially abundant bacterial families detected in the samples. SBM: soybean meal

To evaluate the effect of feed ingredients on chicken caecal microbiota, 24-h in vitro fermentations were performed using SBM or wheat and compared to a control without substrate. Microbial fermentation was assessed by endpoint pH and SCFA quantification in the supernatant, and by pressure measurements at 2, 4, 6 and 24 h (Fig. 2; Supplementary Fig. S3). Acetate, propionate and butyrate are important energy sources for enterocytes and contribute to the regulation of intestinal inflammation and protection against pathogens (Liu et al. 2021). These three SCFAs generally represent more than 95% of the total SCFAs produced by the microbiota and are typically found in an average ratio of 60:25:15 (Canani et al. 2011). This ratio was confirmed across all tested conditions (Fig. 2), with acetate being the most abundant SCFA (Supplementary Table S4). Both SBM and wheat significantly increased acetate and butyrate production compared to the control (Fig. 2A and C), which was consistent with the higher-pressure values observed during incubation (Supplementary Fig. S3A), confirming that both substrates were fermented in vitro. Wheat produced a stronger increase in all quantified SCFAs (*p* < 0.003), which is probably linked to its higher content in fermentable carbohydrates compared to SBM.

In addition to the quantification of SCFA production by the caecal microbiota, a 16S DNA metabarcoding study was carried out to assess bacterial diversity (Supplementary Table S5). As illustrated in Fig. 2D, the abundance profile of the top 17 bacteria families detected in the control caecal contents are consistent with previous findings (Segura-Wang et al. 2021). Indeed, one-third of the bacterial community is represented by *Lachnospiraceae*, followed by the *Ruminococcaceae* (18%), *Peptostreptococcaceae* (13%) and *Lactobacillaceae* (11%) families, respectively. This composition was significantly influenced depending on the substrate tested (Fig. 2D). Bray–Curtis clustering (Fig. 2E) showed clear separation between the control microbiota and the microbiota exposed to wheat, while the bacterial populations exposed to SBM remained closer to those of the control group. This observation is consistent with the significant decrease of pH (from 6.88 ± 0.06 to 6.53 ± 0.10) induced by wheat at the end of fermentation (*p* < 0.001; Supplementary Fig. S3B). Principal coordinate analysis indicated that wheat had a stronger effect on bacterial diversity than SBM, with 81.4% of the variation explained by differences between groups rather than variation within replicates (*p* = 0.001).

Indeed, 13 out of the top 17 bacterial families detected were identified as significantly discriminant between dietary groups by LEfSe analysis (Fig. 2F), highlighting a pronounced effect of feed exposure on gut microbiota structure. In particular, *Lactobacillaceae* were identified as a major biomarker associated with wheat, displaying a higher relative abundance in the wheat group (24.3 ± 2.9%) compared with both the control (11.3 ± 0.6%) and SBM (17.1 ± 1.3%) groups (Supplementary Table S6). Similarly, *Enterococcaceae* were associated with wheat, with a 4.6-fold higher relative abundance compared with the control group (LDA score = 3.98; *p* = 0.023), suggesting a selective response of aerotolerant lactic acid bacteria to this feed component. In contrast, *Enterobacteriaceae* were identified as discriminant for SBM (LDA score = 4.27; *p* = 0.012). This family can be associated with dysbiosis and includes important zoonotic foodborne bacteria such as *Salmonella* (Sharma et al. 2025), although complementary analyses would be required to confirm this association*.* Notably, *Ruminococcaceae* and *Peptostreptococcaceae* families also displayed high LDA scores (LDA > 4), in association with the control condition, suggesting that these resident *Firmicutes* are particularly responsive to feed component. Overall, these results indicate that wheat and SBM modified gut microbiota composition, generating distinct bacterial profiles relative to the control baseline community.

### Effect of Rovabio™ Advance treatment of SBM and wheat on chicken caecal microbiota

**Fig. 3:**
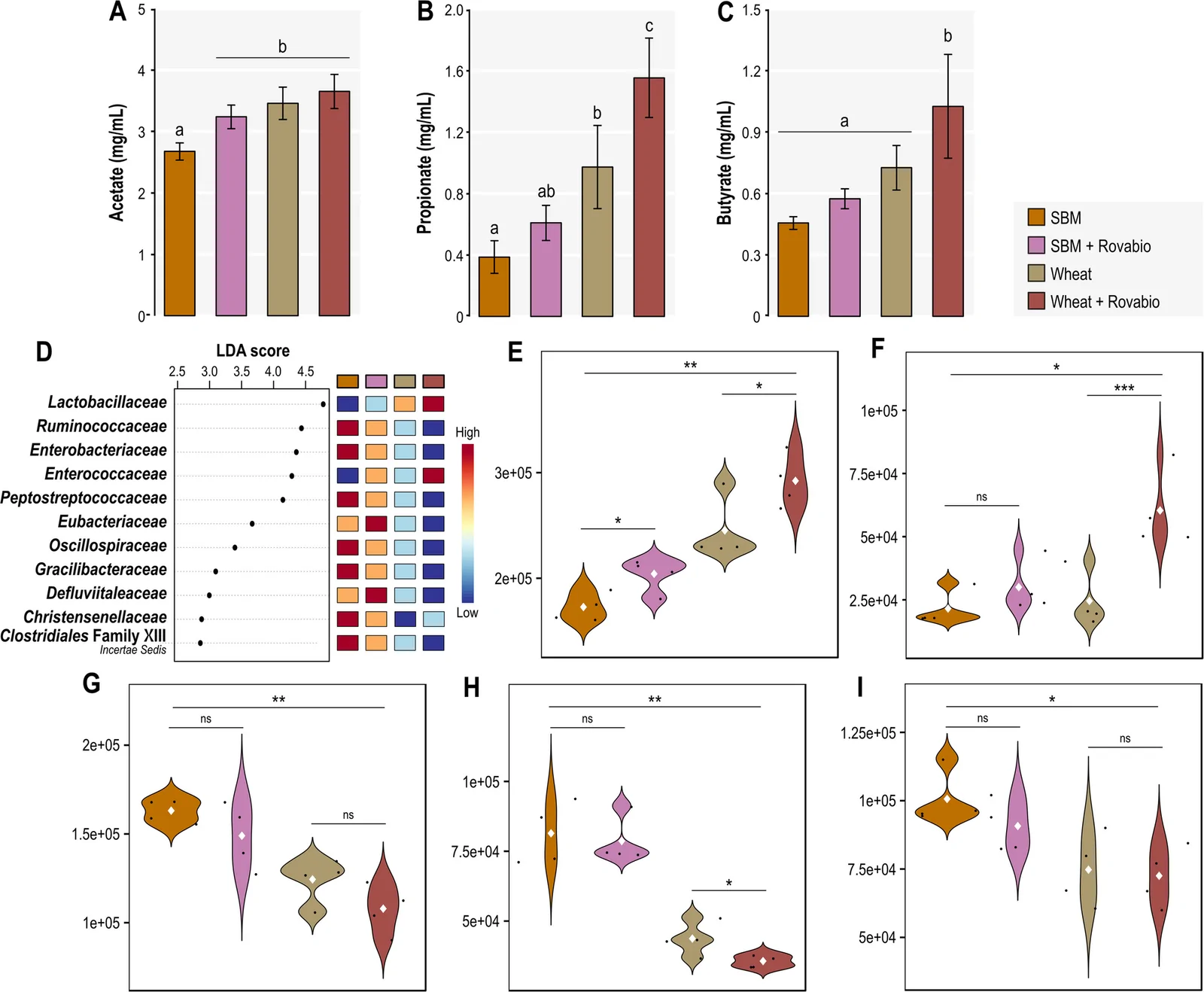
Effect of Rovabio™ Advance treatment of feed component on chicken caecal microbiota. (**A**–**C**) Concentration of acetate (**A**), propionate (**B**) and butyrate (**C**) in mg/mL in the supernatant after 24-h fermentation. Values are presented as means ± SD (biological replicates, *n* = 4). Statistical analyses were performed using one-way ANOVA and Tukey post hoc. Groups indicated by different letters are statistically different (*p* < 0.05). (**D**) Linear discriminant analysis (LDA) effect size (LEfSe) comparison of differentially abundant bacterial families detected in the samples. (E–I) Filtered counts of highly discriminant bacterial families *Lactobacillaceae* (**E**), *Enterococcaceae* (**F**), *Ruminococcaceae* (**G**), *Enterobacteriaceae* (**H**) and *Peptostreptococcaceae* (**I**). Statistical analyses were performed using Linear discriminant analysis (LDA) effect size (LEfSe) comparison of differentially abundant bacterial families and Multiple Linear Regression with Covariate Adjustment (MaAsLin2; biological replicates, *n* = 4). ***p < 0.001; **p < 0.01; *p < 0.05. SBM: soybean meal

The effect of Rovabio™ Advance pre-treatment on the chicken caecal microbiota during SBM and wheat fermentation was then investigated. Enzymatic treatment of SBM increased acetate production relative to untreated SBM (*p* = 0.018; Fig. 3A) but did not significantly affect propionate or butyrate levels. In contrast, pre-treatment of wheat induced a significant increase of propionate (*p* = 0.007; Fig. 3B) and butyrate production (*p* = 0.046; Fig. 3C), while acetate concentrations remained unchanged. Together, these findings indicate a stronger functional response to Rovabio™ Advance supplementation in wheat than in SBM.

Regarding microbial composition, LEfSe analysis identified 11 bacterial families as significantly discriminant (*p* < 0.05) between dietary groups (Fig. 3D; Supplementary Table S7). Among them, several taxa displayed high LDA scores, notably *Lactobacillaceae* (Fig. 3E) and *Enterococcaceae* (Fig. 3F), which emerged as major biomarkers associated with Rovabio™ Advance-treated wheat. In contrast, the SBM diet was primarily characterized by higher abundances of *Ruminococcaceae* (Fig. 3G), *Enterobacteriaceae* (Fig. 3H) and *Peptostreptococcaceae* (Fig. 3I). Importantly, Rovabio™ Advance supplementation of SBM did not significantly modify the abundance of these SBM-associated families, whereas its application to wheat resulted in clear shifts in several bacterial groups, consistent with the SCFA profiles. Notably, the increase in *Lactobacillaceae* observed in Rovabio™ Advance-treated wheat suggests an expansion of lactic acid bacteria, which are commonly associated with rapid carbohydrate fermentation (Wang et al. 2021). Conversely, *Enterobacteriaceae*, which include genera comprising opportunistic or zoonotic species (Yin et al. 2025; Sharma et al. 2025), remained predominantly associated with SBM and were not significantly affected by enzymatic treatment (Fig. 3H), although species-level resolution would be required to assess potential health implications.

Overall, these findings demonstrate that Rovabio™ Advance exerts a stronger modulatory effect on the chicken caecal microbiota when applied to wheat than to SBM. This differential response likely reflects the distinct NSP composition of the two feed components. Wheat is rich in arabinoxylans, β-glucans and cellulose, whereas SBM primarily contains pectin together with xyloglucans and galactomannans (Knudsen 2014). Given that Rovabio™ Advance was developed to target cereal NSPs (Plouhinec et al. 2023), the greater microbiota modulation observed during wheat fermentation is consistent with its enzymatic specificity.

### Effect of SBM enzymatic treatment by pectin-active secretomes on chicken caecal microbiota

**Fig. 4:**
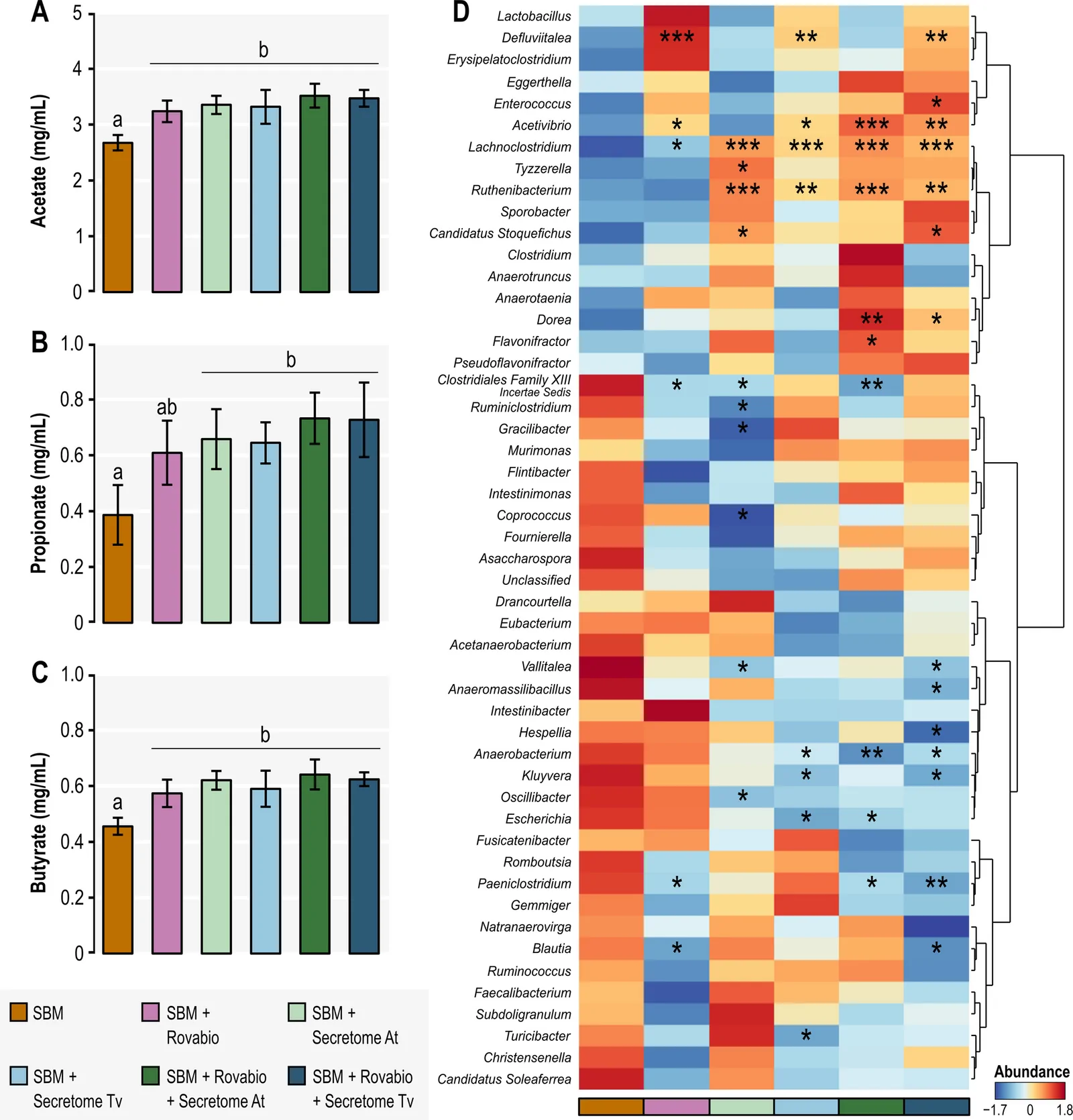
Effect of soybean meal enzymatic treatment on chicken caecal microbiota. (**A**–**C**) Concentration of acetate (**A**), propionate (**B**) and butyrate (**C**) in mg/mL in the supernatant after 24-h fermentation. Values are presented as means ± SD (biological replicates, *n* = 4). Statistical analyses were performed using one-way ANOVA and Tukey post hoc. Groups indicated by different letters are statistically different (*p* < 0.05). (**D**) Heatmap representation of the relative abundance of top 50 bacterial genera detected in the samples. Statistical analyses were performed using Multiple Linear Regression with Covariate Adjustment (MaAsLin2; biological replicates, *n* = 4), using SBM as reference. ***p < 0.001; **p < 0.01; *p < 0.05. SBM: soybean meal

Considering the pectin-rich composition of SBM, its impact on chicken caecal microbiota was further evaluated following enzymatic treatment with Rovabio™ Advance and pectin-active fungal secretomes, applied individually or in combination. As shown in Fig. 4, all enzymatic treatments significantly increased acetate and butyrate production compared with untreated SBM (*p* < 0.02). Propionate production was also significantly increased following enzymatic treatment, rising from 0.39 ± 0.11 mg/mL to 0.73 ± 0.09 mg/mL when SBM was pre-treated with a combination of Rovabio™ Advance and fungal secretomes (*p* < 0.03; Supplementary Table S4). Interestingly, treatment with fungal secretomes alone resulted in SCFA levels comparable to those obtained with Rovabio™ Advance, despite a three-fold lower enzyme load (Supplementary Table S2), highlighting their strong enzymatic efficiency toward SBM polysaccharides. As neither Rovabio™ Advance nor the fungal secretomes alone affected SCFA production in the absence of substrate (Supplementary Table S4), the observed increases can be attributed to fermentation of oligosaccharides released during SBM enzymatic degradation. This suggests that the generated degradation products selectively stimulated specific bacterial populations.

Consistent with this hypothesis, 16S DNA metabarcoding analysis revealed marked shifts in bacterial composition following enzymatic treatment (Fig. 4D). At the genus level, Rovabio™ Advance significantly increased *Acetivibrio* and *Lachnoclostridium* (*p* < 0.05), two taxa previously associated with complex carbohydrate fermentation (Šuchová and Puchart 2025; Deng et al. 2025), while reducing *Blautia*, *Paeniclostridium* and members of *Clostridiales* family XIII (*p* < 0.05). In contrast, fungal secretomes induced broader compositional changes. When used alone, both fungal secretomes reduced *Ruminiclostridium*, *Gracilibacter*, *Coprococcus*, *Valitalea* and *Oscillibacter* (*p* < 0.05). The *A. terreus* secretome specifically increased *Lachnoclostridium* and *Ruthenibacterium* (*p* < 0.001), as well as *Tyzzerella* and *Candidatus Stoquefichus* (*p* < 0.05). Similarly, the *T. versatilis* secretome promoted *Lachnoclostridium* and *Ruthenibacterium* (*p* < 0.001), together with *Defluviitalea* (*p* = 0.004) and *Acetivibrio* (*p* = 0.015), while reducing *Anaerobacterium*, *Kluyvera*, *Turicibacter* and *Escherichia* (*p* < 0.05). Combined treatment further modulated the microbiota, notably increasing *Enterococcus*, *Dorea* and *Flavonifractor* (*p* < 0.05). As no significant changes in pH were observed compared with untreated SBM (Supplementary Fig. S3B), these compositional shifts are more likely associated with the availability of enzymatically released substrates rather than pH-driven effects.

## Discussion

In this study, we investigated the effect of enzymatic treatment of SBM — a protein-rich agricultural co-product widely used in the feed industry — on animal intestinal health. Experiments were performed using human and porcine intestinal epithelial cell (IEC) models, as no commercially available chicken IEC line currently displays fully functional tight junctions. Although the chicken clonal line CHIC-8E11 has been used to study host–pathogen interactions (Ali et al. 2020; Kolenda et al. 2021), it neither secretes inflammatory cytokines nor forms tight junctions. In contrast, Caco-2 and IPEC-J2 are well-characterized monogastric IEC models that exhibit functional tight junctions and cytokine production, making them suitable surrogates for investigating chicken intestinal health (John et al. 2017; Marks et al. 2022). Using TEER measurements and IL-8 quantification, we showed that SBM did not impair epithelial integrity or induce inflammatory responses in vitro under the tested conditions. Enzymatic pre-treatment with commercial cocktails or fungal secretomes did not further modify these parameters, suggesting that the treatments did not generate IEC-damaging compounds under our experimental conditions. To our knowledge, this is the first study assessing the effects of SBM, with or without enzymatic pre-treatment, on animal IECs in vitro. Indeed, previous work has primarily investigated the antioxidant properties and biological activities of soybean protein extracts in Caco-2 cells (Zhu et al. 2002; Zhang et al. 2018).

In this context, in vitro analyses of chicken caecal microbiota were conducted, including SCFA quantification and 16S metabarcoding, to gain insight into the effects of feed composition and enzymatic treatment on microbiota homeostasis. Comparison of wheat and SBM showed that wheat exerted a stronger influence on bacterial fermentation, as evidenced by increased production of acetate, propionate and butyrate. Consistent with previous reports, wheat was also associated with a higher relative abundance of the beneficial gut bacterial family *Lactobacillaceae* (Borda-Molina et al. 2021; Kim et al. 2022). In contrast, microbial fermentation of SBM was associated with *Enterobacteriaceae*, suggesting an environment that might be propitious to the growth of opportunistic pathogens, although further analyses would be required to identify the species involved.

Further comparison of SBM and wheat following enzymatic treatment with Rovabio™ Advance indicated a stronger efficiency in modulating microbial fermentation of wheat, notably through increased SCFA production, as previously reported both in vitro and in vivo (Yacoubi et al. 2016, 2018). Treatment of wheat was also associated with an enrichment of *Lactobacillaceae* and *Enterococcaceae*, two families that include members previously linked to improved gut barrier function and immune responses in broiler chickens (Zhang et al. 2025). In contrast, the impact of Rovabio™ Advance on SBM fermentation and bacterial diversity was comparatively limited. These differences are consistent with the enzymatic profile of Rovabio™ cocktails, which is mainly enriched in hemicellulases, cellulases, and debranching enzymes (Guais et al. 2008) but contain fewer pectin-degrading enzymes targeting SBM polysaccharides (Plouhinec et al. 2025).

Treatment of SBM with pectin-active fungal secretomes, used alone or in combination with Rovabio™ Advance, significantly increased acetate, propionate and butyrate production by the chicken caecal microbiota in vitro. Butyrate is commonly associated with microbiota homeostasis in chickens, particularly because of its protective effects against opportunistic pathogens and its role in maintaining epithelial integrity through the promotion of tight junction formation (Singh et al. 2023). It has also been associated with increased body weight gain due to its influence on adipocyte development, making it a metabolite of particular interest in poultry production (Ismael et al. 2025). While SBM hydrolysis with Rovabio™ Advance alone did not enhance propionate production compared with untreated SBM, supplementation with fungal secretomes resulted in a significant increase. This response may be partly associated with the enrichment of bacterial genera such as *Tyzzerella*, *Dorea* and *Flavonifractor*, which have previously been linked with propionate production by the gut microbiota (Polansky et al. 2016; Bernard et al. 2024; Scott et al. 2025). Given the strong pectinolytic activity of *A. terreus* and *T. versatilis* secretomes reported previously (Plouhinec et al. 2024), these results support the contribution of pectin-degrading enzymes to improving the fermentability and prebiotic potential of SBM.

It should be acknowledged that microbiota analyses based on SCFA quantification and 16S metabarcoding primarily reveal associations between dietary interventions, microbial composition and metabolic outputs. While such approaches provide valuable insights into microbiota dynamics, they do not allow direct inference of causal relationships between specific bacterial taxa, enzymatic activities and host responses (Koh and Bäckhed 2020; Basic et al. 2022). Addressing these limitations will require more integrative strategies combining complementary functional and multi-omics approaches (Arıkan and Muth 2023). In particular, metaproteomic analyses could provide direct evidence of the CAZymes mobilised by chicken gut microbiota in response to SBM hydrolysates, as previously demonstrated in the human gut microbiota following exposure to resistant starch (Maier et al. 2017). Such approaches may also facilitate the discovery of novel CAZymes within the chicken gut microbiota (Jiang et al. 2024). In parallel, a more detailed characterisation of SBM hydrolysates would improve our understanding of the contribution of released mono- and oligosaccharides to the observed microbial responses. Indeed, studies on human and pig faecal microbiota have shown that the prebiotic effects of pectin strongly depend on its structural features and origin (Onumpai et al. 2011; Guo et al. 2023), while certain pectin-derived compounds may also influence pathogen virulence (Jimenez et al. 2019). Given that *A. terreus* and *T. versatilis* secretomes efficiently degrade the pectin backbone, releasing uronic acids and rhamnose residues (Plouhinec et al. 2024), the increased SCFA production observed herein may be linked to the presence of oligogalacturonides and rhamnogalacturonan-derived oligosaccharides. Fractionation of SBM hydrolysates by size or charge, as previously performed for enzymatically-treated wheat (Yacoubi et al. 2016), would therefore represent a valuable next step to better define the prebiotic potential of SBM-derived carbohydrates.

Finally, previous in vivo studies have demonstrated that enzymatically-treated SBM improves nutritional value, growth performance and physiological parameters in broiler chickens (Kocher et al. 2002; Meng et al. 2005; Jiang et al. 2022), and that fermented SBM can reduce pathogen colonisation and improve gut microbiota health (Jazi et al. 2019; Li et al. 2020). Together, these observations support the relevance of tailoring enzyme solutions to target SBM carbohydrates, while underscoring the need for integrative in vivo multi-omics and functional studies to fully elucidate the molecular mechanisms involved.

## Conclusions

Overall, this study provides the first in vitro evaluation of the effects of enzymatically-treated SBM on chicken intestinal health. Using animal IEC models, we showed that enzyme-treated SBM did not induce epithelial inflammation nor affect epithelial integrity in the conditions tested. In contrast, in vitro fermentation experiments demonstrated that SBM significantly modulated caecal microbial activity and community composition, as reflected by changes in SCFA production and bacterial abundance. Enzymatic treatment of SBM, particularly with pectin-active fungal secretomes, enhanced microbial fermentation and promoted the production of health-associated SCFAs such as butyrate and propionate. Together, these findings highlight the potential of targeted enzymatic strategies to modulate SBM fermentation by the chicken gut microbiota and support intestinal health. They also provide a foundation for future in vivo and functional studies to optimise the nutritional and microbiological properties of SBM in poultry diets, and to tailor commercial enzymatic cocktails for greater effectiveness on both cereals and oilseed meals.

## Supplementary Information

Below is the link to the electronic supplementary material

ESM 1 (XLSX 34.0 KB)

ESM 2 (PDF 809 KB)

## Data Availability

All data generated or analysed during this study are included in this published article and its supplementary information files. Illumina sequencing raw data are accessible on the NCBI under the reference PRJNA1465736.
