# Supplementary material for: Impact of soybean meal enzymatic treatment on chicken caecal microbiota and intestinal epithelial cells
Source: Appl Microbiol Biotechnol. 2026 Jun 23;110:263. doi: 10.1007/s00253-026-13925-8 (PMC13542025; doi:10.1007/s00253-026-13925-8)
Supplement: Supplementary file 1 [file 253_2026_13925_MOESM2_ESM.pdf]

Impact of soybean meal enzymatic treatment on chicken caecal microbiota and intestinal epithelial cells

Applied Microbiology and Biotechnology

Lauriane Plouhinec[1,4], Marc Maresca[2], Julie Saget[3], Virginie Neugnot[4], Estelle Devillard[3], Jean-Guy Berrin[1,*], Mickael Lafond[1,*]

[1]Aix-Marseille Univ, INRAE, BBF, Biodiversité et Biotechnologie Fongiques, 13009 Marseille, France

[2]Aix Marseille Univ, CNRS, Centrale Marseille, iSM2, 13013 Marseille, France

[3]Adisseo France S.A.S., European R&I Centre ELISE, 69190 Saint-Fons, France

[4]Adisseo France S.A.S, CINAbio, INSA Toulouse, 31400 Toulouse, France

*corresponding authors:

Jean-Guy Berrin (jean-guy.berrin@inrae.fr)

Mickael Lafond (michael.lafond@univ-amu.fr)

Supplementary information

SUPPLEMENTARY TABLE S1. Experimental conditions used for in vitro intestinal cell lines assays. Quantities of SBM are expressed as mg of dry matter in the reaction. Quantities of Rovabio™ Advance and secretomes are expressed as mg of total proteins in the reaction. (*) This condition corresponds to the negative control containing the reaction buffer only (i.e., 50 mM sodium acetate pH 5.2). SBM: soybean meal; At: *A. terreus*; Tv: *T. versatilis.*

Condition	SBM	Rovabio™ Advance	Secretome At	Secretome Tv
**Control***	-	-	-	-
**Rovabio**	-	0.9 mg	-	-
**Secretome At**	-	-	0.3 mg	-
**Secretome Tv**	-	-	-	0.3 mg
**Rovabio + Secretome At**	-	0.9 mg	0.3 mg	-
**Rovabio + Secretome Tv**	-	0.9 mg	-	0.3 mg
**SBM**	75 mg	-	-	-
**SBM + Rovabio**	75 mg	0.9 mg	-	-
**SBM + Secretome At**	75 mg	-	0.3 mg	-
**SBM + Secretome Tv**	75 mg	-	-	0.3 mg
**SBM + Rovabio + Secretome At**	75 mg	0.9 mg	0.3 mg	-
**SBM + Rovabio + Secretome Tv**	75 mg	0.9 mg	-	0.3 mg

SUPPLEMENTARY TABLE S2. Experimental conditions used for in vitro caecal fermentations. Quantities of SBM and wheat are expressed as mg of dry matter in the reaction. Quantities of Rovabio™ Advance and secretomes are expressed as mg of total proteins in the reaction. (*) This condition corresponds to the negative control containing the reaction buffer only (i.e., 50 mM sodium acetate pH 5.2). SBM: soybean meal; At: *A. terreus*; Tv: *T. versatilis.*

Condition	SBM	Wheat	Rovabio™ Advance	Secretome At	Secretome Tv
**Control***	-	-	-	-	-
**Rovabio**	-	-	0.9 mg	-	-
**Secretome At**	-	-	-	0.3 mg	-
**Secretome Tv**	-	-	-	-	0.3 mg
**Rovabio + Secretome At**	-	-	0.9 mg	0.3 mg	-
**Rovabio + Secretome Tv**	-	-	0.9 mg	-	0.3 mg
**SBM**	75 mg	-	-	-	-
**Wheat**	-	75 mg	-	-	-
**SBM + Rovabio**	75 mg	-	0.9 mg	-	-
**SBM + Secretome At**	75 mg	-	-	0.3 mg	-
**SBM + Secretome Tv**	75 mg	-	-	-	0.3 mg
**SBM + Rovabio + Secretome At**	75 mg	-	0.9 mg	0.3 mg	-
**SBM + Rovabio + Secretome Tv**	75 mg	-	0.9 mg	-	0.3 mg

SUPPLEMENTARY TABLE S3. Two-way ANOVA and Tukey post-hoc analysis of IPEC-J2 TEER values. Transepithelial electric resistance was determined after 12 h exposure on IPEC-J2 cells (biological replicates, n = 3). "Untreated" corresponds to the reference control (i.e., cells in culture media). SBM hydrolysates were tested at different dilutions in the culture media (from 1:80 to 1:10) to assess the dose effect. Statistical analyses were performed using RStudio, as described in the Methods section. Groups indicated by different letters are statistically different ($p < 0.05$). DON: deoxynivalenol; SBM: soybean meal; At: *A. terreus*; Tv: *T. versatilis.*

Refer to Supplementary Excel file SI_Plouhinec et al._2026_Table S3

SUPPLEMENTARY TABLE S4. SCFA concentrations in the caecal fermentation supernatants. Quantification was done by GC-MS after 24h fermentation, as described in the Methods section. Means and standard deviations were calculated from biological replicates (n = 4). Statistical analyses were performed using one-way ANOVA and Tukey post-hoc. Groups indicated by different letters are statistically different (p < 0.05). (*) This condition corresponds to the negative control containing the reaction buffer only (i.e., 50 mM sodium acetate pH 5.2). SBM: soybean meal; At: *A. terreus*; Tv: *T. versatilis.*

	**Acetate (mg/mL)**		**Propionate (mg/mL)**		**Butyrate (mg/mL)**	
**Sample**	Mean	SD	Mean	SD	Mean	SD
**Control***	1.41 ^{a}	0.10	0.17 ^{a}	0.06	0.26 ^{a}	0.03
**Rovabio**	1.49 ^{a}	0.10	0.19 ^{a}	0.06	0.27 ^{a}	0.03
**Secretome At**	1.51 ^{a}	0.11	0.18 ^{a}	0.07	0.28 ^{a}	0.03
**Secretome Tv**	1.43 ^{a}	0.16	0.17 ^{a}	0.07	0.26 ^{a}	0.04
**Rovabio + Secretome At**	1.61 ^{a}	0.02	0.20 ^{a}	0.07	0.29 ^{a}	0.05
**Rovabio + Secretome Tv**	1.60 ^{a}	0.09	0.20 ^{a}	0.07	0.28 ^{a}	0.05
**Wheat**	3.46 ^{c}	0.26	0.97 ^{d}	0.27	0.73 ^{c}	0.11
**Wheat + Rovabio**	3.65 ^{c}	0.28	1.55 ^{e}	0.26	1.03 ^{d}	0.25
**SBM**	2.67 ^{b}	0.14	0.39 ^{ab}	0.11	0.46 ^{ab}	0.03
**SBM + Rovabio**	3.24 ^{c}	0.19	0.61 ^{bc}	0.11	0.57 ^{bc}	0.05
**SBM + Secretome At**	3.35 ^{c}	0.16	0.66 ^{bcd}	0.11	0.62 ^{bc}	0.03
**SBM + Secretome Tv**	3.32 ^{c}	0.30	0.64 ^{bc}	0.07	0.59 ^{bc}	0.06
**SBM + Rovabio + Secretome At**	3.52 ^{c}	0.21	0.73 ^{cd}	0.09	0.64 ^{bc}	0.05
**SBM + Rovabio + Secretome Tv**	3.47 ^{c}	0.15	0.73 ^{cd}	0.13	0.62 ^{bc}	0.02

SUPPLEMENTARY TABLE S5. Taxonomic classification statistics. This table contains data provided by BaseClear; the service provider responsible for performing the metabarcoding analysis of the chicken caecal contents. For each sample, the table displays the number of classified and unclassified reads, alongside alpha diversity indices such as richness (Chao) and diversity (Simpson and Shannon) index (Xia and Sun 2023). All values represent means calculated from biological replicates (n = 4). (*) This condition corresponds to the negative control containing the reaction buffer only (i.e., 50 mM sodium acetate pH 5.2). SBM: soybean meal; At: *A. terreus*; Tv: *T. versatilis.*

Sample	Classified reads	Unclassified reads	Chao index	Simpson index	Shannon index
**Control***	11732	27	229.5	0.0479	5.51
**Rovabio**	11745	24	242.8	0.0492	5.49
**Secretome At**	11712	25	235.8	0.0482	5.51
**Secretome Tv**	11624	32	235.1	0.0488	5.49
**Rovabio + Secretome At**	11641	21	238.3	0.0464	5.55
**Rovabio + Secretome Tv**	11767	28	238.0	0.0467	5.54
**SBM**	11679	25	231.4	0.0512	5.34
**Wheat**	11568	22	202.2	0.0666	4.96
**SBM + Rovabio**	11750	22	222.0	0.0526	5.24
**SBM + Secretome At**	11691	20	225.9	0.0544	5.24
**SBM + Secretome Tv**	11619	20	220.5	0.0524	5.26
**SBM + Rovabio + Secretome At**	11598	24	218.8	0.0528	5.25
**SBM + Rovabio + Secretome Tv**	11633	23	223.5	0.0521	5.26

SUPPLEMENTARY TABLE S6. Linear discriminant analysis effect size (LEfSe) comparison of differentially abundant bacterial families detected in the samples. This table presents the relative abundance of the top 17 bacterial families detected in the control and in the microbiota exposed to SBM or wheat. Means and standard deviations were calculated from biological replicates (n = 4). (*) This condition corresponds to the negative control containing the reaction buffer only (i.e., 50 mM sodium acetate pH 5.2). SBM: soybean meal.

**Sample**	**Control***		**SBM**		**Wheat**		**LDA score**	***p*-value**
Family	Mean	SD	Mean	SD	Mean	SD		
Lachnospiraceae	34.06%	1.53%	33.86%	1.46%	39.15%	3.31%	4.42	0.059
Ruminococcaceae	18.14%	0.95%	16.13%	0.63%	12.34%	1.25%	4.47	0.007
Peptostreptococcaceae	13.34%	0.55%	9.95%	0.97%	7.42%	1.31%	4.48	0.007
Lactobacillaceae	11.30%	0.58%	17.09%	1.30%	24.33%	2.99%	4.82	0.007
Enterobacteriaceae	6.85%	1.01%	8.05%	1.11%	4.34%	0.58%	4.27	0.012
Erysipelotrichaceae	4.17%	0.42%	3.04%	0.22%	2.98%	0.40%	3.79	0.023
Eubacteriaceae	2.58%	0.27%	2.11%	0.12%	1.56%	0.13%	3.72	0.009
unclassified Clostridiales	2.57%	0.21%	2.01%	0.24%	1.45%	0.43%	3.76	0.017
Clostridiaceae	1.84%	0.14%	1.68%	0.09%	1.48%	0.18%	3.28	0.063
Gracilibacteraceae	1.12%	0.15%	0.67%	0.08%	0.55%	0.08%	3.52	0.012
Enterococcaceae	0.52%	0.11%	2.13%	0.67%	2.44%	1.07%	3.98	0.023
Christensenellaceae	0.47%	0.04%	0.41%	0.08%	0.27%	0.03%	3.02	0.018
Defluviitaleaceae	0.41%	0.11%	0.38%	0.03%	0.25%	0.03%	2.92	0.024
Eggerthellaceae	0.38%	0.05%	0.33%	0.03%	0.23%	0.08%	2.89	0.077
Oscillospiraceae	0.29%	0.10%	0.61%	0.25%	0.16%	0.09%	3.36	0.017
Clostridiales Family XIII *Incertae Sedis*	0.25%	0.09%	0.28%	0.01%	0.15%	0.04%	2.81	0.058
Paenibacillaceae	0.19%	0.03%	0.15%	0.04%	0.02%	0.01%	2.93	0.015

SUPPLEMENTARY TABLE S7. Linear discriminant analysis (LDA) effect size (LEfSe) comparison of differentially abundant bacterial families detected in the samples after treatment with Rovabio™ Advance. This table presents the relative abundance of the top 17 bacterial families detected in the microbiota exposed to SBM or wheat, with and without treatment with Rovabio™ Advance. Means and standard deviations were calculated from biological replicates (n = 4). SBM: soybean meal.

**Sample**	**SBM**		**SBM + Rovabio**		**Wheat**		**Wheat + Rovabio**		**LDA score**	***p*-values**
Family	Mean	SD	Mean	SD	Mean	SD	Mean	SD		
Lachnospiraceae	33.85%	1.46%	33.20%	1.94%	39.15%	3.31%	34.65%	0.74%	4.47	0.072
Ruminococcaceae	16.13%	0.63%	14.74%	1.82%	12.34%	1.25%	10.71%	1.37%	4.44	0.009
Peptostreptococcaceae	9.95%	0.97%	8.97%	0.91%	7.42%	1.30%	7.20%	1.07%	4.15	0.020
Lactobacillaceae	17.08%	1.30%	20.20%	1.58%	24.33%	2.99%	29.01%	2.51%	4.78	0.004
Enterobacteriaceae	8.05%	1.11%	7.78%	0.82%	4.34%	0.58%	3.54%	0.21%	4.36	0.006
Erysipelotrichaceae	3.04%	0.22%	2.92%	0.13%	2.98%	0.40%	2.56%	0.42%	3.39	0.142
Eubacteriaceae	2.11%	0.12%	2.15%	0.37%	1.56%	0.13%	1.24%	0.32%	3.67	0.010
unclassified Clostridiales	2.01%	0.24%	1.73%	0.32%	1.45%	0.43%	1.28%	0.30%	3.57	0.074
Clostridiaceae	1.68%	0.09%	1.62%	0.30%	1.48%	0.18%	1.54%	0.14%	3.02	0.442
Gracilibacteraceae	0.67%	0.08%	0.60%	0.11%	0.55%	0.08%	0.42%	0.05%	3.10	0.023
Enterococcaceae	2.13%	0.67%	2.97%	0.99%	2.44%	1.07%	5.99%	1.52%	4.29	0.017
Christensenellaceae	0.41%	0.08%	0.35%	0.05%	0.27%	0.03%	0.27%	0.07%	2.88	0.022
Defluviitaleaceae	0.38%	0.03%	0.44%	0.10%	0.25%	0.03%	0.24%	0.02%	3.00	0.009
Eggerthellaceae	0.33%	0.03%	0.34%	0.15%	0.22%	0.08%	0.24%	0.08%	2.78	0.146
Oscillospiraceae	0.61%	0.25%	0.55%	0.24%	0.16%	0.09%	0.11%	0.02%	3.40	0.010
Clostridiales Family XIII *Incertae Sedis*	0.28%	0.01%	0.22%	0.06%	0.15%	0.04%	0.14%	0.05%	2.86	0.018
Corynebacteriaceae	0.17%	0.03%	0.15%	0.02%	0.13%	0.05%	0.10%	0.01%	2.54	0.065

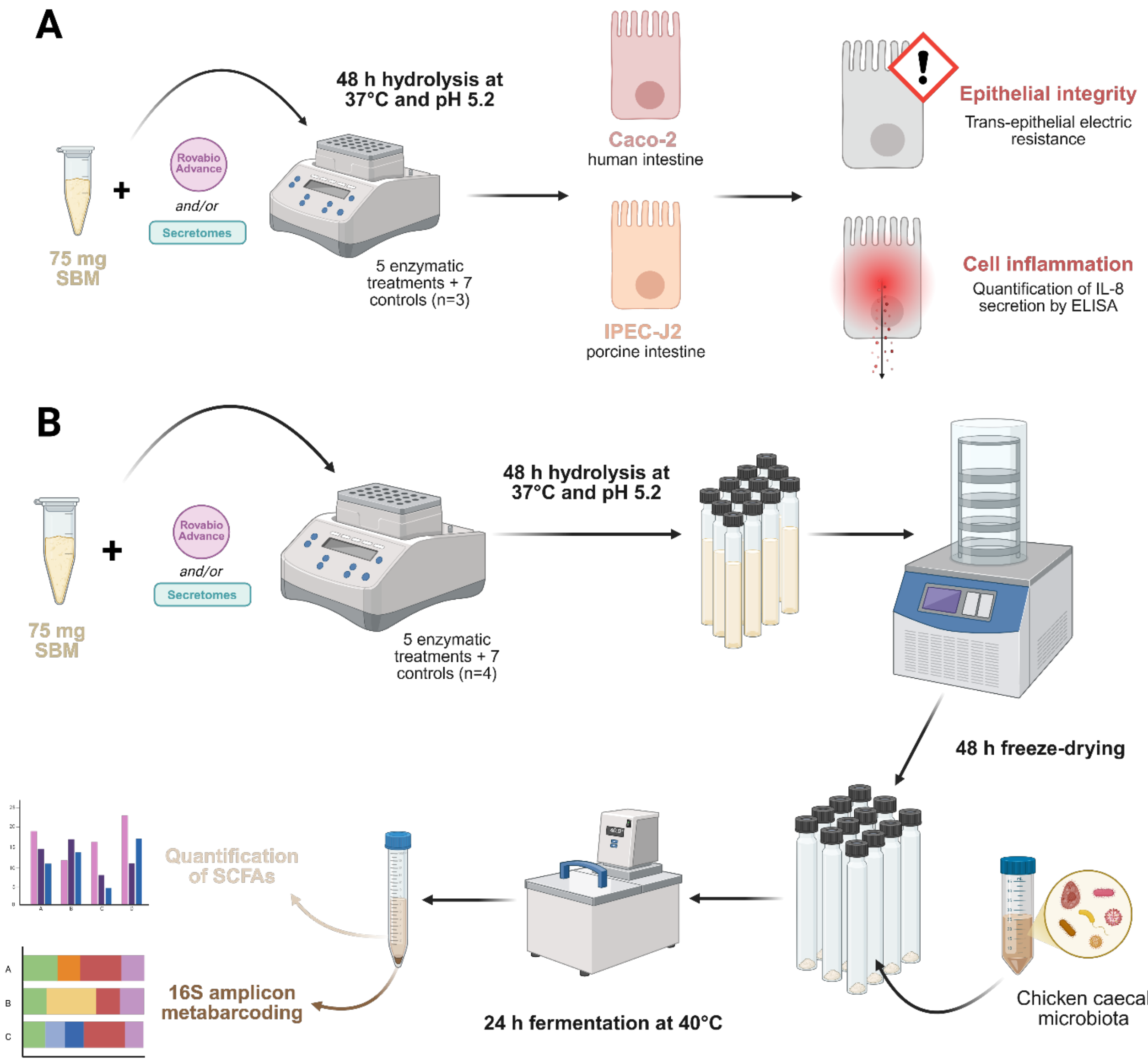

SUPPLEMENTARY FIGURE S1. Overview of the experimental design for the in vitro evaluation of SBM on intestinal health. This figure was designed using BioRender software app.biorender.com. SBM: soybean meal; SCFAs: short-chain fatty acids.

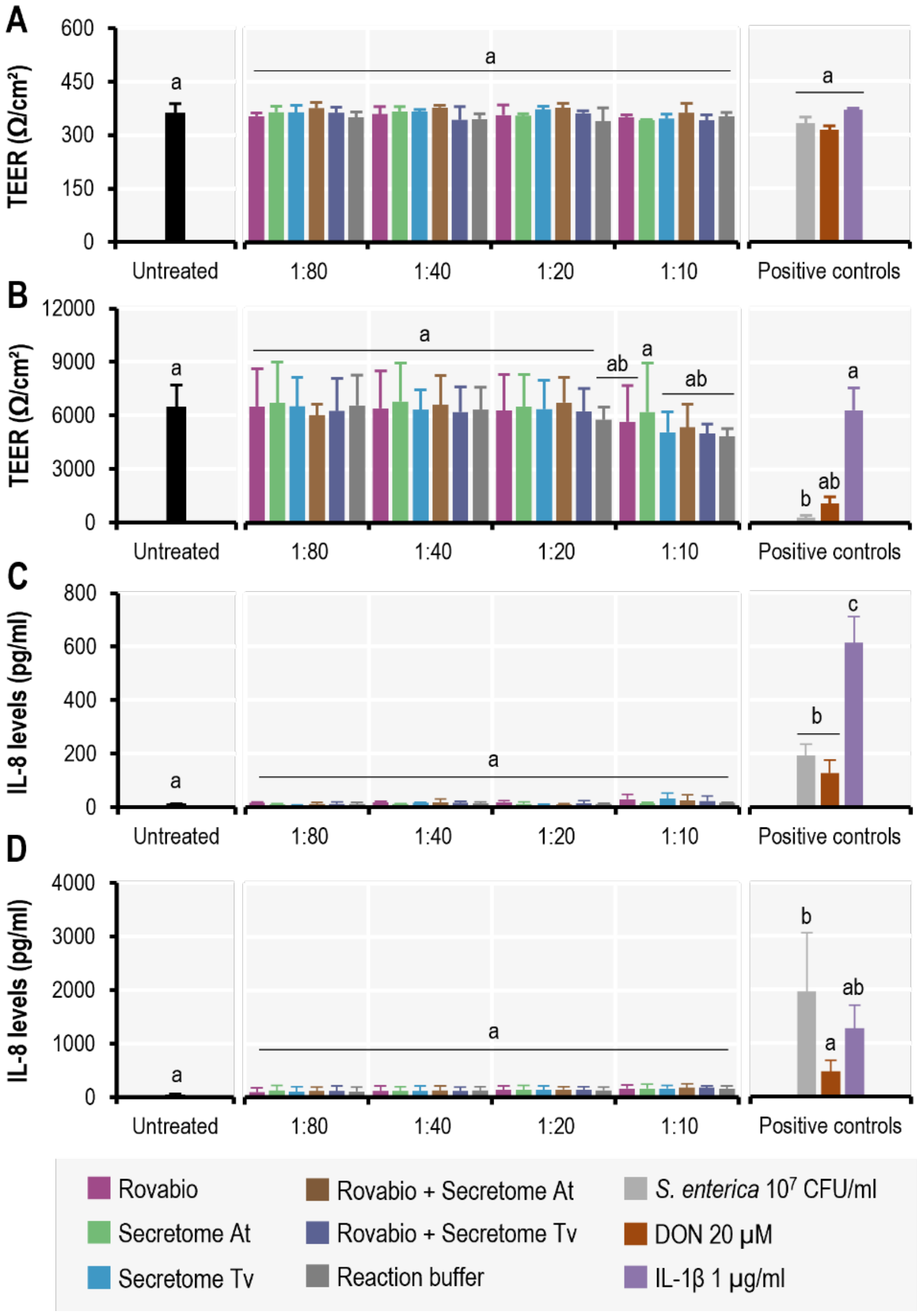

SUPPLEMENTARY FIGURE S2. Effect of enzymatic cocktails on in vitro epithelial integrity and cytokine production. (A,B) Trans-epithelial electric resistance after 12 h exposure on **(A)** Caco-2 model and **(B)** IPEC-J2 model. **(C,D)** IL-8 secretion after 12 h exposure on **(C)** Caco-2 model and **(D)** IPEC-J2 model. Values are presented as means ± SD (biological replicates, n = 3). "Untreated" corresponds to the reference control (i.e., cells in culture media). Samples were tested at different dilutions in the culture media (from 1:80 to 1:10), to assess the dose effect. Statistical analyses were performed using two-way ANOVA and Tukey post-hoc. Groups indicated by different letters are statistically different ($p < 0.05$).

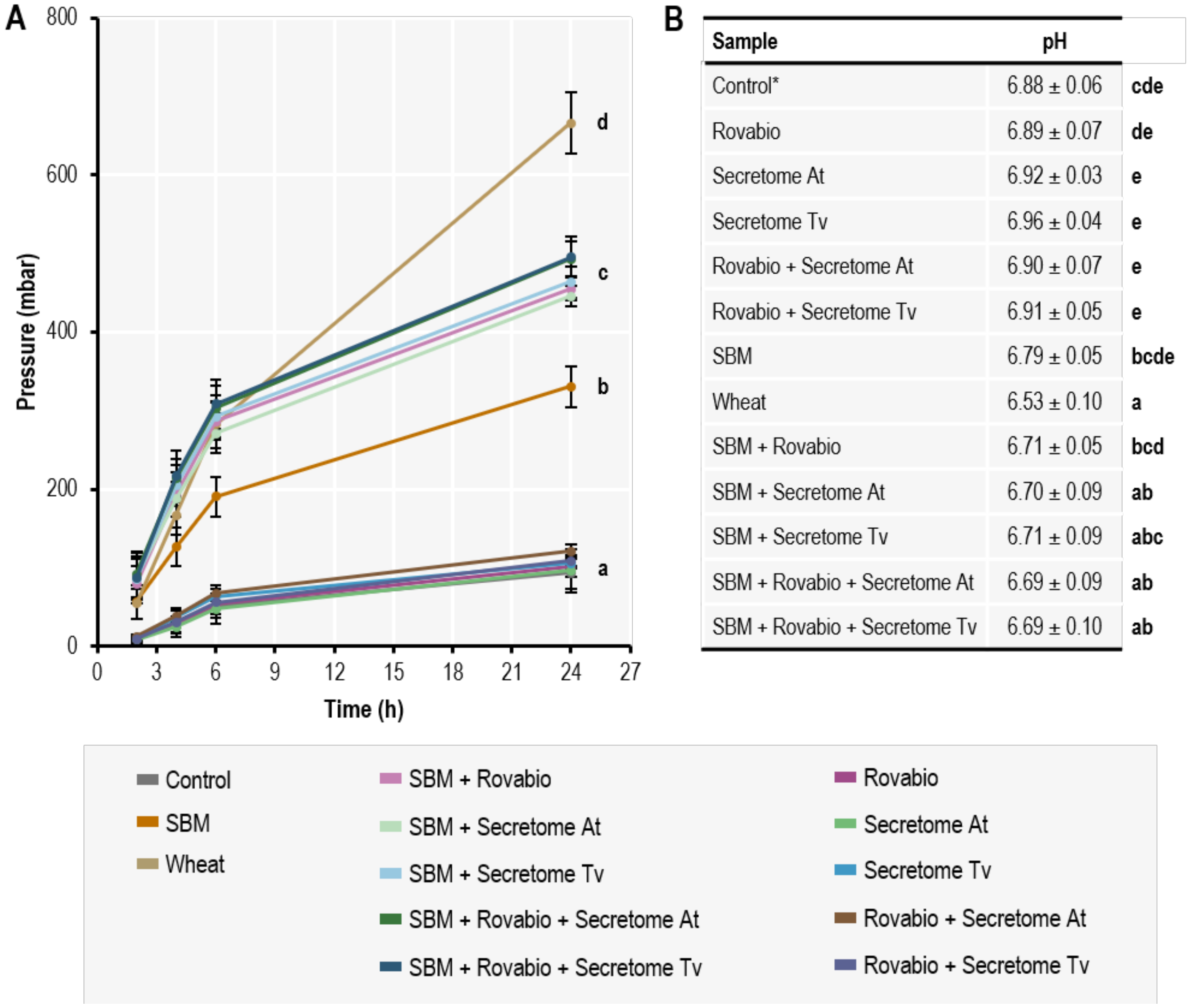

Sample	pH	
Control*	6.88 ± 0.06	cde
Rovabio	6.89 ± 0.07	de
Secretome At	6.92 ± 0.03	e
Secretome Tv	6.96 ± 0.04	e
Rovabio + Secretome At	6.90 ± 0.07	e
Rovabio + Secretome Tv	6.91 ± 0.05	e
SBM	6.79 ± 0.05	bcde
Wheat	6.53 ± 0.10	a
SBM + Rovabio	6.71 ± 0.05	bcd
SBM + Secretome At	6.70 ± 0.09	ab
SBM + Secretome Tv	6.71 ± 0.09	abc
SBM + Rovabio + Secretome At	6.69 ± 0.09	ab
SBM + Rovabio + Secretome Tv	6.69 ± 0.10	ab

SUPPLEMENTARY FIGURE S3. Monitoring of in vitro caecal fermentations. (A) Pressure measurement (mbar) at 2, 4, 6 and 24 h of fermentation. **(B)** pH of the fermentation supernatants after 24 h. Values are presented as means ± SD (biological replicates, n = 4). Statistical analyses were performed using one-way ANOVA and Tukey post-hoc. Groups indicated by different letters are statistically different ($p < 0.05$). (*) This condition corresponds to the negative control containing the reaction buffer only (i.e., 50 mM sodium acetate pH 5.2). SBM: soybean meal; At: *A. terreus*; Tv: *T. versatilis.*

References

Xia Y, Sun J (2023) Alpha Diversity. In: Xia Y, Sun J (eds) Bioinformatic and statistical analysis of microbiome data: from raw sequences to advanced modelling with QIIME 2 and R. Springer International Publishing, Cham, pp 289–333
